# Descriptive analysis of depression among adolescents in Huangshi, China

**DOI:** 10.1186/s12888-023-04682-3

**Published:** 2023-03-16

**Authors:** Xiaozhi Zhang, Yueming Yan, Zhuofan Ye, Jumin Xie

**Affiliations:** 1 Psychological children’s Ward, Mental Health Center of Huangshi, 435111 Hubei, China; 2 Hubei Key Laboratory of Renal Disease Occurrence and Intervention, Medical School, Hubei Polytechnic University, Guilin north road, No 16, Xialu district, Huangshi, 435003 Hubei China; 3 Department of Neurology, Clinical College, Zunyi Medical University, Zuiyi, 563000 Guizhou P. R. China

**Keywords:** Adolescent, Descriptive analysis, Depression, Risk factors

## Abstract

**Background:**

More adolescents suffered from depressive disorder, and what was worse, the morbidity increased annually. The situation was getting worse during COVID-19 pandemic. The prevalence of depression among adolescents in China has increased a lot due to social and economic development, family-associated reasons, academic stress, interpersonal relationships, and so on.

**Objective:**

This study aimed to determine the prevalence, gender differences, risk factors, and abnormal illness behaviors of depression among adolescents in Huangshi, China.

**Methods:**

A descriptive analysis was conducted based on the data from clinical interviews and self-reports by the patients. Depression was assessed and diagnosed using the DSM-5 Diagnostic and Statistical Manual of Mental Disorders.

**Results:**

Depression was most frequently seen in 674 patients with mental illnesses (282, 41.84%). The male-to-female ratio was 1:2.44, and their age ranged from 9 to 18. The majority of patients are in high school (261/282, 92.55%), and the highest morbidity occurred at 16 years. More cases were diagnosed in urban than in rural areas. Genetic factors, school violence, academic stress, sleep disorders, and family-related factors were essential factors leading to depression among adolescents. Most patients had sleep disorders (84.75%). In family-related factors, left-behind children and unrecognized/misunderstood by their families were prominently diagnosed with depression. A large portion of individuals with depression felt apathetic, solitary, and sluggish and were unable to study, work, and live normally (212/282, 75.18%); they even committed suicide or attempted suicide (228/282, 80.85%) and inflicted self-harm (146/282, 51.77%).

**Conclusions:**

An increasing trend of depression has been observed since 2018, especially in 2021. This depression has led to suicide or suicidal attempts and self-harm, reflecting the severity of mental health among adolescents in Huangshi. Therefore, this study aimed to draw the attention of society, families, and schools to the importance of mental health among adolescents, providing guidance and references for the prevention, diagnosis, and treatment of young depressive disorders in China.

## Introduction

Depression, also known as Major Depressive Disorder, is one of the most common mental disorders, characterized by a persistent feeling of sadness, loss of interest, and, in extreme cases, suicidal thoughts [1]. Based on the Global Health Data Exchange, approximately 3.8% of the population, comprising 280 million people worldwide, suffered from depression, which was a major contributor to the global disease burden [2, 3]. According to the World Health Organization (WHO) report, the GBD 2019 Mental Disorders Collaborators state that depression is becoming a leading cause of disability [2].

To date, the causes of depression are still uncertain, largely due to biological, genetic, environmental, and psychological factors [4]. Depression affects almost 4–5% of adolescents worldwide annually, and the treatment burden is heavy [5]. In the United States and China, the situation was much worse, with the prevalence rates of adolescent depressive symptoms at 12% and 24.6%, respectively [6, 7]. People with depression are increasing worldwide, especially during the COVID-19 pandemic [8, 9], with a higher incidence in 2021 than in 2019 and 2018, demonstrating the grave consequences of COVID-19. Depression is considered a huge burden of disease worldwide and is projected to rank first by 2030 [3]. A person with depression often experiences discrimination and stigma, which influences all aspects of their life, such as school or work performance, relationships with family and friends, and their ability to participate in society [10].

Social, family, school, dwelling environment, psychological, and genetic factors all contribute to depression [11]. More adolescent females developed depression than male adolescents [2, 12], and the gender difference might correlate with their hormones, for instance estradiol and progesterone sexual hormones [13, 14]. According to the China National Mental Health Development Report (2019–2020), the incidence rate of depression in high school was as high as 40%, correlating with academic stress and social interaction [15]. Sleep deprivation impacts physical health, mood, behaviors, and academic performance among adolescents, which is an essential factor in acquiring depressive disorder [16, 17].

Globally, an increasing number of children and adolescents are being left at home when their parents migrate. In a meta-analysis, results showed that compared to non-left-behind children, left-behind children and adolescents had a 52% increased risk of depression, a 70% increased risk of suicidal ideation, and an 85% increased risk of anxiety [18]. According to the statistics of the Ministry of Civil Affairs of China, by the end of August 2018, the number of left-behind children in China had reached 6.97 million, illustrating the serious situation of left-behind children in China [19]. Besides, single-parent families were also important in the development of adolescent depression. Moreover, school violence played an important role in the formation of depressive episodes [20].

In this study, an epidemiological analysis of the collected cases was performed and summarized based on the time of visit, gender, dwelling environment, and associated risk factors. These findings would provide a reference and clinical guidance for the prevention and treatment of depression among adolescents in China.

## Materials and methods

### Cases collection

A total of 674 cases of mental disorders in individuals aged 18 or younger were collected from October 1, 2017 to July 31, 2022.

### Inclusion criteria

The DSM-5 Diagnostic and Statistical Manual of Mental Disorders was used as the diagnostic criteria [21].

We recruited patients aged 18 years or younger with available case files and meeting DSM-5 criteria. Exclusion criteria included patients who sought medical attention but did not have a depressive disorder, with incomplete information, and who did not meet the DSM-5 criteria for diagnosis.

### Abnormal illness behaviors

Due to the *Severe Psychiatric Diseases Management and Treatment Norms 2018* published by China’s Health Commission (in Chinese), abnormal illness behaviors can be divided into nine subtypes: M1, previously received psychiatric treatment; M2, impulsively hurting people, destroying things, or running away from home for no reason; M3, acting strangely and appearing unkempt or naked in public; M4, often laughing out loud for no reason or saying something that does not make sense; M5, suspicious/everyone around is targeting or persecuting him; M6, overly excited, nonstop talkative, active, trouble-making, or running around; M7, apathetic, solitary, sluggish, and unable to study, work, live normally; M8, committed suicide and/or attempts; and M9, self-harm [22].

### Assessment of causes

The assessment of genetic factors was based on a self-report and clinical interview of the patient who suffered from depression within three generations of the paternal and maternal lineages. School violence and academic stress were due to their complaints about school life, academic pressure, and violence. Family-related factors and sleep disorders were confirmed through self-report and talking to their parents.

## Results

### Constituent ratio of depression among adolescents in Huangshi

**Fig. 1:**
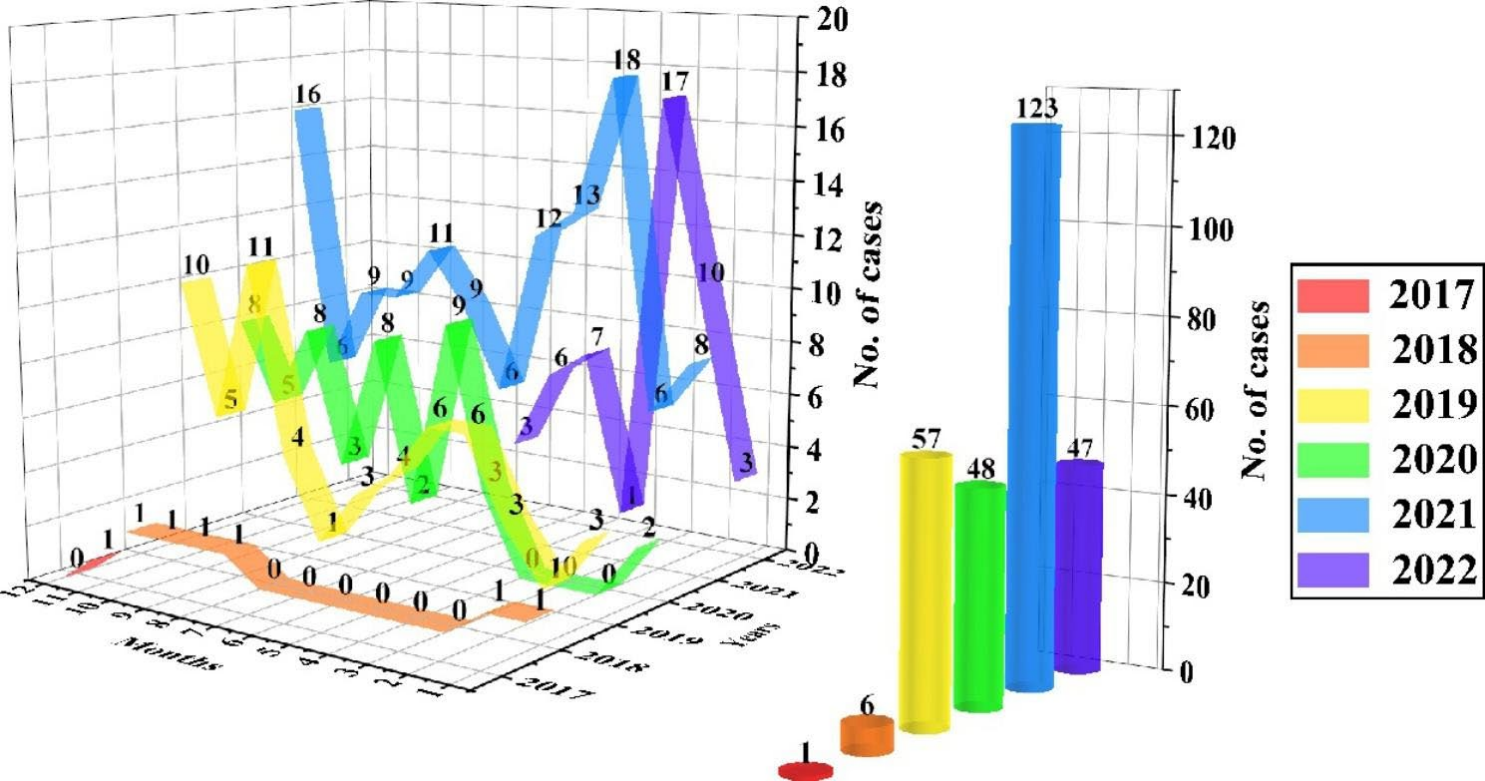
Cases distribution between October 1, 2017 to July 31, 2022

A total of 282 cases of depression were diagnosed, accounting for 41.84% of the 674 participants. The distribution of cases by different years and months is listed in (Fig. 1). The dramatic increase occurred in 2021, accounting for 43.62% of the total 282 cases. The most vulnerable month was March (Fig. 1). Partial data were available in 2017 and 2022; however, the morbidity increased annually. The proportion of patients with depression who sought medical treatment in 2021 was 2.16- and 2.56-fold higher than in 2019 and 2020, respectively (data not shown).

### Gender differences

**Fig. 2:**
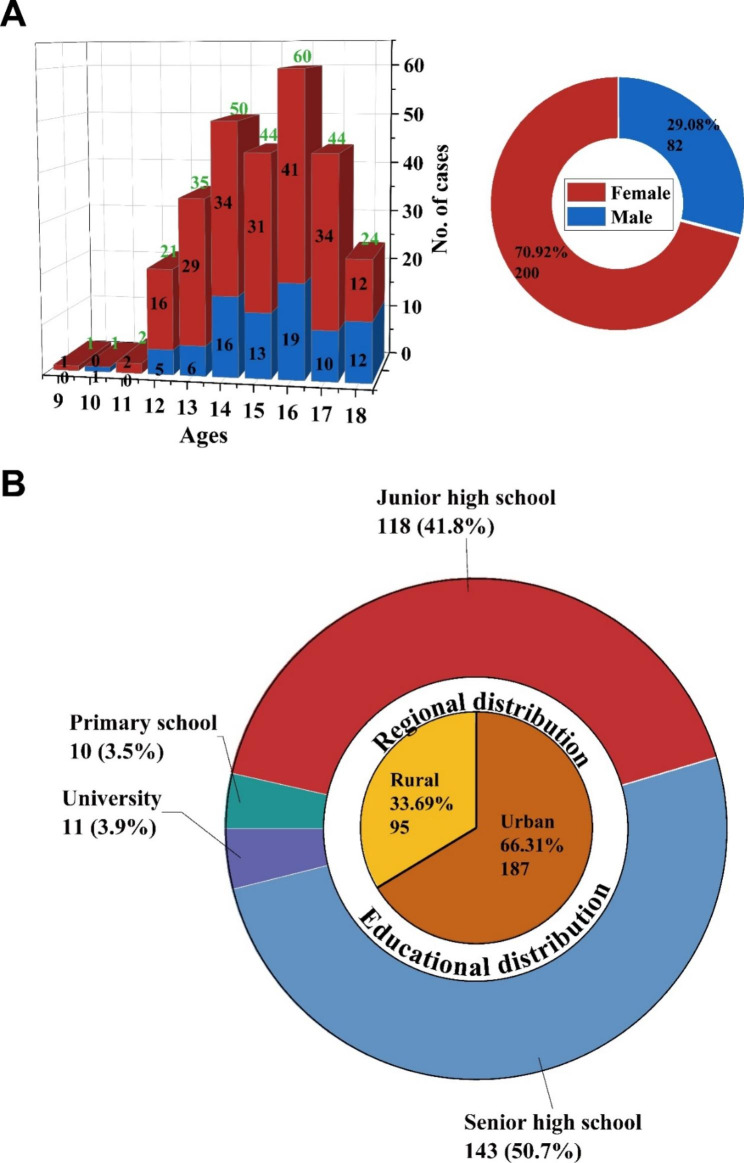
General information of participants. (**A**) Gender distribution and ratio. (**B**) Regional and educational distribution

The 282 cases comprised 82 boys and 200 girls (Fig. 2A). The gender ratio and age ranges were 1:2.44 and 9–18 (average, 15.06 ± 1.81), respectively (Fig. 2A). The highest incidence was observed in 16-year-olds (Fig. 2A).

### Regional and educational distribution

Among the 282 cases, 187 lived in urban areas (66.31%), and the remaining 95 cases lived in rural areas (Fig. 2B). Educational levels were also analyzed, showing that the majority of patients were in high school (261/282, 92.55%) (Fig. 2B). The peak emergence of depression occurred in senior high school patients (143/282, 50.71%), surpassing one-half of the study population (Fig. 2B).

### Possible factors causing depression

**Fig. 3:**
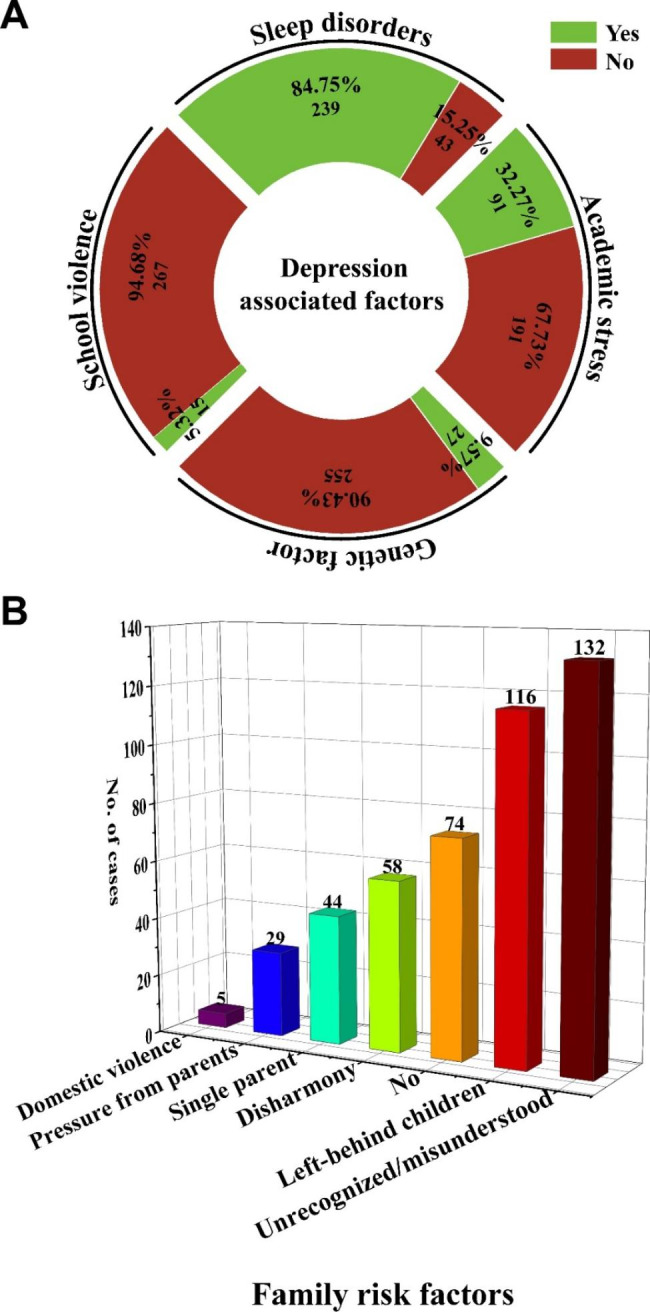
Possible causes of depression. (**A**) Depression associated factors. (**B**) Family risk factors

In this study, 9.57% (27/282) of patients had genetic predispositions (Fig. 3A). School violence contributes to 5.32% (15/282) of depressed participants, whereas the situation was worse overseas (Fig. 3A). About 32.27% (91/282) of cases reported that their depression was influenced by academic stress (Fig. 3A). A large proportion (239/282, 84.75%) of cases suffered from sleep disorders, however, it cannot be determined whether these are the results or causes (Fig. 3A).

About 73.76% (208/282) of cases reported that the cause for their depression was associated with their families. When we analyzed the possible family-related factors, the top two reasons were unrecognized/misunderstood by the family and left-behind children, which accounted for 46.81% (132/282) and 41.13% (116/282) (Fig. 3B).

### Abnormal illness behaviors

**Fig. 4:**
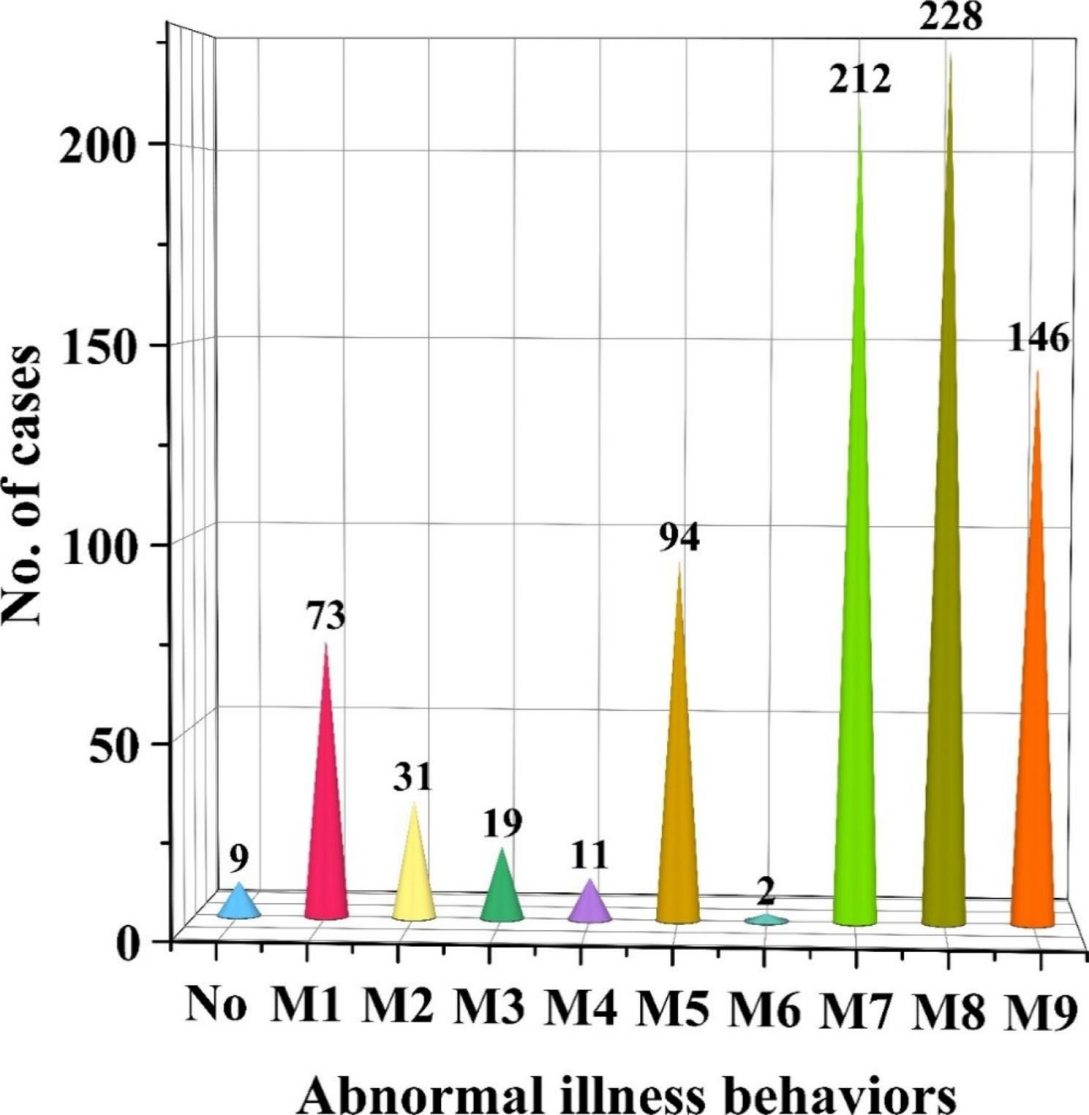
Abnormal illness behaviors

Among the 282 cases, 80.82% (228/282) committed suicide or attempted it (M8) (Fig. 4). The second most common abnormal illness behavior in this study was apathetic, solitary, sluggish, and unable to study, work, or live normally (M7, 212, 75.18%), followed by self-harm (M9, 146, 51.77%).

## Discussion

According to data collected by GBD 2019, mental disorders increased by 48.1% between 1990 and 2019, while depression increased by 63.7% [2].

The situation is similar in Huangshi, Hubei Province, China. According to the Huangshi Municipal Bureau of Statistics, the resident population in 2021 was approximately 2.4 million, of which 0.6 million were ≤ 18 years old. Our analysis estimated the weighted prevalence of depression among adolescents in Huangshi to be between 10% and 15%.

In this study, 282 patients were included, with more girls suffering from depression than boys. More cases developed in March, which might correlate with the start of the term. Based on age analysis, the number of cases increased at the ages of 14 and 16 years, which was relevant to entrance exams to junior and senior high schools. The onset age was concentrated between 13 and 17 years in our analysis. Of the 282 cases, 261 (92.55%) came from high school. The majority of patients in this study came from high school, which strongly suggested an intensive correlation between academic stress and depression occurrence.

Moreover, the incidence of depression was found to be higher in urban than rural areas. This might be related to the economic development, recognition, and convenience in the urban area. Then, we analyzed the possible causes of depression in adolescents. Genetic factors, school violence, academic stress, sleep disorders, and family-related factors were all associated with the occurrence of depression. Among the associated factors, academic stress and family-related factors were strongly correlated with depression in adolescents.

In this study, 84.75% of cases admitted to having sleep disturbances; however, determining the causal relationship between sleep disorders and depression was difficult. Genetic factors accounted for approximately one tenth of the patients in this study. School violence has by no means a negligible role in depression; however, the situation is mild in China compared to overseas. A large proportion of patients were related to family reasons, especially left-behind children and those who were unrecognized or misunderstood by their families.

The nine abnormal illness behaviors classified by China’s Health Commission better guide us to recognize the destructive behaviors among adolescents with depression. The top one abnormal illness behavior in depression was committing suicide and/or attempts (M8). Additionally, most participants with depression harbored apathy, solitude, sluggishness, an inability to study, work, or live normally (M7), and self-harm (M9). Shockingly, the abnormal illness behaviors among depressed adolescent cases in our survey were much worse than expected. These results indicate the severity of depression among adolescents in China.

Moreover, affected by traditional Chinese culture, people were reluctant to see a psychiatrist or be regarded as psychotic. Therefore, we speculate that depression among adolescents might be worse than our analysis suggests due to the difficulty of detection and humiliation of patients. However, more patients were seeking medical treatment in 2021 than in 2018, reflecting a positive trend in cognition toward depression. Because of social discrimination, cognitive deficiency, and stigma, patients disguise and repress their emotions until they break out. Due to the immaturity of young people, they are often ignored. Once diagnosed, the prognosis is often grim. Our study found that the prevalence of depression among adolescents is increasing.

## Conclusion

In this study, the prevalence of depression among adolescents in Huangshi was investigated. The most vulnerable group was high school students aged 13–18 years. Academic stress and family-related factors were critical causes of depression among adolescents. More girls were diagnosed with depressive disorder than boys, which might be correlated with their sensitive emotions and sexual hormones. Sleep disorder was extremely common among adolescents. Most diagnosed patients felt apathetic, solitary, sluggish, unable to study, work, or live normally, and even self-harm and attempted suicide. Depression among adolescents in China is worse and more severe than expected.

## Data Availability

The processed data was available in the paper, and raw data is freely serviced from corresponding author.
